# Default Mode Network Structural Integrity and Cerebellar Connectivity Predict Information Processing Speed Deficit in Multiple Sclerosis

**DOI:** 10.3389/fncel.2019.00021

**Published:** 2019-02-11

**Authors:** Giovanni Savini, Matteo Pardini, Gloria Castellazzi, Alessandro Lascialfari, Declan Chard, Egidio D’Angelo, Claudia A. M. Gandini Wheeler-Kingshott

**Affiliations:** ^1^Department of Physics, University of Milan, Milan, Italy; ^2^Department of Neurosciences, Rehabilitation, Ophthalmology, Genetics and Maternal and Child Health, University of Genoa, Genoa, Italy; ^3^Ospedale Policlinico S. Martino, Genoa, Italy; ^4^Department of Electrical, Computer and Biomedical Engineering, University of Pavia, Pavia, Italy; ^5^NMR Research Unit, Queen Square MS Centre, Department of Neuroinflammation, Institute of Neurology, University College London, London, United Kingdom; ^6^National Institute for Health Research, University College London Hospitals, Biomedical Research Centre, London, United Kingdom; ^7^Department of Brain and Behavioral Sciences, University of Pavia, Pavia, Italy; ^8^Brain Connectivity Center, IRCCS Mondino Foundation, Pavia, Italy; ^9^Brain MRI 3T Mondino Research Center, IRCCS Mondino Foundation, Pavia, Italy

**Keywords:** default mode network (DMN), cerebellum, multiple sclerosis (MS), symbol digit modalities test (SDMT), connectomics, tractography, diffusion weighted imaging (DWI), magnetic resonance imaging (MRI)

## Abstract

Cognitive impairment affects about 50% of multiple sclerosis (MS) patients, but the mechanisms underlying this remain unclear. The default mode network (DMN) has been linked with cognition, but in MS its role is still poorly understood. Moreover, within an extended DMN network including the cerebellum (CBL-DMN), the contribution of cortico-cerebellar connectivity to MS cognitive performance remains unexplored. The present study investigated associations of DMN and CBL-DMN structural connectivity with cognitive processing speed in MS, in both cognitively impaired (CIMS) and cognitively preserved (CPMS) MS patients. 68 MS patients and 22 healthy controls (HCs) completed a symbol digit modalities test (SDMT) and had 3T brain magnetic resonance imaging (MRI) scans that included a diffusion weighted imaging protocol. DMN and CBL-DMN tracts were reconstructed with probabilistic tractography. These networks (DMN and CBL-DMN) and the cortico-cerebellar tracts alone were modeled using a graph theoretical approach with fractional anisotropy (FA) as the weighting factor. Brain parenchymal fraction (BPF) was also calculated. In CIMS SDMT scores strongly correlated with the FA-weighted global efficiency (GE) of the network [GE(CBL-DMN): ρ = 0.87, *R*^2^ = 0.76, *p* < 0.001; GE(DMN): ρ = 0.82, *R*^2^ = 0.67, *p* < 0.001; GE(CBL): ρ = 0.80, *R*^2^ = 0.64, *p* < 0.001]. In CPMS the correlation between these measures was significantly lower [GE(CBL-DMN): ρ = 0.51, *R*^2^ = 0.26, *p* < 0.001; GE(DMN): ρ = 0.48, *R*^2^ = 0.23, *p* = 0.001; GE(CBL): ρ = 0.52, *R*^2^ = 0.27, *p* < 0.001] and SDMT scores correlated most with BPF (ρ = 0.57, *R*^2^ = 0.33, *p* < 0.001). In a multivariable regression model where SDMT was the independent variable, FA-weighted GE was the only significant explanatory variable in CIMS, while in CPMS BPF and expanded disability status scale were significant. No significant correlation was found in HC between SDMT scores, MRI or network measures. DMN structural GE is related to cognitive performance in MS, and results of CBL-DMN suggest that the cerebellum structural connectivity to the DMN plays an important role in information processing speed decline.

## Introduction

Multiple sclerosis (MS) is an inflammatory, demyelinating, and neurodegenerative disease of the central nervous system and the most frequent non-traumatic cause of permanent neurological disability in young adults (Inglese, 2006). Cognitive impairment occurs in about 50% of MS patients independently of physical disability (Chiaravalloti and DeLuca, 2008; Dineen et al., 2009; Hulst et al., 2013), but the mechanisms underlying this are still poorly understood and roles of the specific brain networks in MS are not clearly identified. Information processing speed is known to be one of the core deficits in MS (Chiaravalloti and DeLuca, 2008) and the SDMT (Smith, 1982) assesses this. When compared with other cognitive measures the SDMT has been shown to better distinguish MS patients and HCs (Strober et al., 2009; Benedict et al., 2017) and correlate with MRI metrics (Christodoulou et al., 2003; Stankiewicz et al., 2011; Rao et al., 2014; Benedict et al., 2017). Moreover, the SDMT has been tested against extensive batteries of neuropsychological tests specifically developed to detect general cognitive impairment within an MS population like the Neuropsychological Battery for MS (Rao et al., 1991) and the Minimal Assessment of Cognitive Functioning in MS (Benedict et al., 2002): the SDMT has been proposed as a sentinel test to detect general cognitive impairment in MS with 91% sensitivity and 60% specificity (Parmenter et al., 2007; Van Schependom et al., 2014).

Information processing speed, and SDMT performance, is associated with activity in the default mode network (DMN) (Rocca et al., 2010; Sumowski et al., 2010; Forn et al., 2013; Bonavita et al., 2015), which deactivates when cognitively demanding tasks are performed (Buckner et al., 2008; Broyd et al., 2009). However, the DMN’s role in MS-related cognitive impairment is not clear and the structural changes leading to dysfunction are not fully captured yet.

In MS, previous studies mostly focused on functional connectivity of individual DMN cortical regions or on structural alterations of individual white matter (WM) bundles connecting the DMN nodes (Rocca et al., 2010; Bonavita et al., 2011, 2016; Weier et al., 2014, 2015; Wojtowicz et al., 2014; Zhou et al., 2014). However, given the widespread nature of MS pathology, a global approach for the assessment of the DMN structural network integrity may provide a more meaningful explanation of its performance and functioning.

Network science enables us to assess global network features, including properties such as efficiency, connectedness, modularity (Bullmore and Sporns, 2009; Rubinov and Sporns, 2010; Kaiser, 2011), all which are abnormal in people with MS (Gamboa et al., 2014; Shu et al., 2016). In the motor network, network efficiency have been shown to correlate more closely than conventional whole brain and regional MRI measures with disability (Pardini et al., 2015). We believe that there is an unmet need to assess the contribution of the DMN structural network, as an integrated system, to cognition in MS and to assess whether the DMN network properties capture cognitive impairment for future clinical translation.

Brain regions classically known to be involved in the DMN are the precuneus/posterior cingulate cortex, medial frontal cortex, middle temporal gyri and angular gyri. Some reports also show that the DMN functional network extends to include cerebellar nodes (CBL-DMN) (Habas et al., 2009; Krienen and Buckner, 2009; Stoodley and Schmahmann, 2010; Buckner et al., 2011; Castellazzi et al., 2018a,b). Measures of network integrity and topology will differ depending on which brain regions are included (Kaiser, 2011), and associations with clinical outcomes may also be affected if functionally relevant regions are omitted. While the cerebellum was classically thought of as a key region underlying sensorimotor function only, it’s role in cognition has been increasingly recognized (Ramnani, 2006; Strick et al., 2009; Tedesco et al., 2011; D’Angelo and Casali, 2013; Koziol et al., 2014; Sokolov et al., 2017) and it has been proven to be involved in SDMT functions (Forn et al., 2011). Moreover, it has been shown that MS lesions in the cerebellum and cortico-cerebellar pathways can result in cognitive deficits (Weier et al., 2015) and there is clear evidence of cerebellar involvement in other central nervous system diseases, such as Alzheimer’s, Parkinson’s, and autism (Fatemi et al., 2012; Wu and Hallett, 2013; Jacobs et al., 2018).

The purpose of this study was to investigate the role of the DMN in MS-related cognitive impairment considering also its structural connections with the cerebellum. We used a network approach to assess whether or not structural alterations, as assessed using diffusion weighted MRI scans, might explain differences in information processing speed. We investigated whether the inclusion of cortico-cerebellar loops within the model of the DMN-CBL network can better explain the derangement of information processing functions caused by MS pathology. Noting the recent proposal of a “network collapse” hypothesis in MS, suggesting that as structural damage reaches a critical threshold detectable symptoms of cognitive impairment become apparent (Schoonheim et al., 2015; Shu et al., 2016), we also examined DMN network efficiency in CIMS and CPMS MS patients.

## Materials and Methods

### Subjects and Clinical Assessment

For this study 68 consecutive patients with relapse-onset MS, attending routine clinical appointments, and a group of 22 HC with no known neurologic or psychiatric condition were enrolled. Patients who had had a relapse or received corticosteroids in the preceding 4 weeks were excluded from the study. The study was approved by the local institutional ethics committee (NRES Committee London – Queen Square) and all subjects provided written informed consent.

All participants had a series of neuropsychological tests, including the SDMT, the HADS-A and HADS-D (Zigmond and Snaith, 1983) and the NART. Expanded disability status scale scores were also assessed.

An SDMT score of 40 points was shown to be the optimal cut-off value to detect general cognitive impairment in a cohort of 359 MS patients (Van Schependom et al., 2014). Within this context, this threshold was used to divide the cohort of MS patients in CIMS (20 subjects) and CPMS (46 subjects) groups. It is important to note that cognitive impairment is here to be interpreted as “cognitive impairment detected with SDMT.”

### MRI Acquisition

All participants underwent a brain 3T MRI scan session using a 3T Philips Achieva MRI scanner (Philips Healthcare, Best, Netherlands) with dual transmit and a 32-channel receive head-coil.

The MRI acquisition protocol included a sagittal high-resolution 3DT1-weighted fast field echo scan (TE = 3.1 ms, TR = 6.9 ms, TI = 824 ms, 1 mm × 1 mm × 1 mm resolution, 256 × 256 acquisition matrix, 180 sagittal slices) and an axial anterior commissure – posterior commissure oriented dual echo PD/T2 scan (TE = 19/85 ms, TR = 3500 ms, 1 mm × 1 mm × 3 mm resolution, FOV = 240 mm × 240 mm). Diffusion weighted images were acquired with a cardiac-gated axial spin echo EPI high angular resolution diffusion imaging scan aligned with the anterior commissure – posterior commissure line (TE = 68 ms, TR ∼ 24 s depending con cardiac rate, 2 × 2 × 2 mm resolution, SENSE factor = 3.1, acquisition matrix = 96 × 112, reconstruction matrix = 112 × 112, 72 slices with no gap, 61 isotropically distributed directions with *b* = 1200 s/mm^2^, 7 volumes with b = 0 s/mm^2^).

### Data Processing

#### Image Pre-processing

Diffusion images were pre-processed with FSL (Jenkinson et al., 2012) (FMRIB Software Library^1^) and MRtrix (Tournier et al., 2012)^2^ tools:

• correction for eddy-current distortions and subsequent diffusion vector realignment were applied;
• non-brain tissue was removed;
• The diffusion tensor was reconstructed and FA maps generated for each subject;
• The fiber orientation density function was evaluated using constrained spherical deconvolution (Tournier et al., 2007) and high-resolution TDIs (Calamante et al., 2010) were generated seeding the whole brain for the generation of 2.5 × 10^6^ tracks. TDI maps provide improved resolution and WM contrast with respect to diffusion tensor maps: these advantages were later exploited for precise positioning of both automatic and manually-drawn regions of interest (ROIs).

Following a pre-defined pipeline (Muhlert et al., 2013), registration steps were computed using NiftyReg tools^3^ in order to register atlas ROIs from MNI152 standard space at 1 mm resolution to the TDI space of each subject. In particular, the MNI152 T1 template was non-linearly registered to the subject-specific T1-weighted space that was in turn rigidly registered to a pseudo-T1 generated by subtracting the PD-weighted from the T2-weighted data, thus providing images with similar WM – gray matter (GM) contrast to the T1 scan. The pseudo-T1 volume is inherently co-registered to the T2-weighted one, which was non-linearly registered to the native diffusion b = 0 space of the subject. A rigid transformation was computed from the native diffusion space to the TDI space of the subject. Inverse transformation matrices were also computed.

For MS subjects, the WM-LL was evaluated on PD/T2 images using JIM (Xinapse Systems^4^). The BPF (Rudick et al., 1999) was evaluated on lesion filled 3DT1 images using LEAP (Chard et al., 2010).

A WM mask was also segmented with SPM^5^ from the 3DT1 applying a 90% threshold.

#### ROIs Selection

A map of the DMN provided in the MNI152 standard space at 2 mm resolution by a previous study (Smith et al., 2009) was binarised and rigidly registered to MNI152 1 mm resolution standard space.

The DMN mask was then divided into eight ROIs corresponding to the nodes of the resting state network and divided between right and left hemispheres. The resulting ROIs correspond to:

• Left and right medial frontal cortex;
• Left and right angular gyrus;
• Left and right precuneus/posterior cingulate cortex;
• Left and right middle temporal gyrus

**FIGURE 1:**
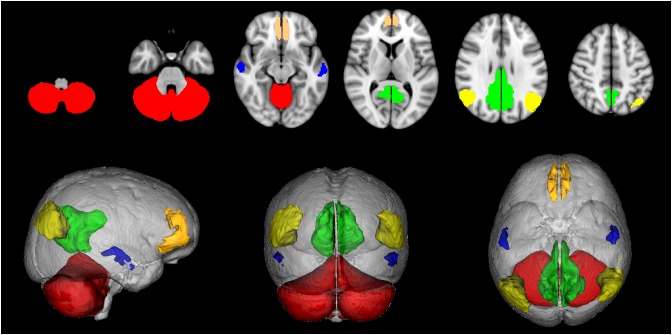
Default mode network (DMN) nodes. Medial frontal cortex (orange), angular gyri (yellow), precuneus/posterior cingulate cortex (green), middle temporal gyri (blue), cerebellum (red).

Since the available DMN map did not include cerebellar nodes a map of the cerebellum was obtained from the SUIT atlas (Diedrichsen, 2006; Diedrichsen et al., 2009), binarised, divided into right and left hemispheres and considered as a further ROI for the study. Thus the CBL-DMN consisted in 10 network nodes (Figure 1).

Further masks were created for tractography seeding, inclusion and exclusion: from the JHU ICBM-DTI-81 atlas, provided with FSL in MNI152 standard space at 1 mm resolution, masks of the superior cerebellar peduncles, of the middle cerebellar peduncles, of the cerebral peduncles and of the corpus callosum were selected.

All binary masks were registered to the TDI space of each HC applying the previously obtained transformation.

Cortical masks in the TDI space of each HC were then dilated toward the inner part of the brain and intersected with the WM mask previously obtained in order to create seed masks containing only voxels at the WM – GM interface.

Exclusion masks were also created for the medulla oblongata to exclude fibers heading toward the spinal cord and for ventricles.

#### Tractography and Network Mask

Probabilistic tractography was performed using MRtrix and the following constraints were adopted for an anatomically plausible reconstruction of all tracts:

• tractography of fibers connecting pairs of homolateral brain cortex ROIs was performed seeding the smaller of the two; the cerebellum, the brainstem, the ventricles, the corpus callosum, and the opposite cerebral hemisphere were considered as exclusion ROIs.
• tractography of fibers connecting pairs of contralateral brain cortex ROIs was performed seeding the corpus callosum; the cerebellum, the brainstem, and the ventricles were considered as exclusion ROIs.
• tractography of fibers connecting contralateral middle temporal gyri was performed seeding the anterior commissure the cerebellum, the brainstem and the ventricles were considered as exclusion ROIs.
• tractography of cerebello-thalamo-cortical fibers projecting from the cerebellum to contralateral brain cortex areas was performed by seeding the superior cerebellar peduncles and including the contralateral brain cortex ROIs (Palesi et al., 2015); with respect to the considered superior cerebellar peduncle the contralateral cerebellum, the homolateral red nucleus, the ventricles, the corpus callosum and the medulla oblongata were considered as exclusion ROIs.
• tractography of cortico-ponto-cerebellar fibers projecting from brain cortex areas to the contralateral cerebellum was performed seeding the cerebral peduncles and including the homolateral brain cortex ROIs and the contralateral middle cerebellar peduncles (Palesi et al., 2017); with respect to the considered cerebral peduncle the homolateral cerebellum, the ventricles, the corpus callosum and the medulla oblongata were considered as exclusion ROIs.

Resulting tracts from 22 HC were registered back to the MNI152 standard space at 1 mm resolution following the inverse registration algorithm and binarised. A population map of each tract was created by adding the tracts in standard space and applying a 50% probabilistic threshold to select only voxels belonging to at least 50% of the subjects.

As per previous works, the resulting masks of the tracts were registered to the FA maps of CIMS and CPMS patients and HC (Pagani et al., 2005; Rocca et al., 2007, 2010) and intersected once again with WM mask. Then, for each subject, the mean and the standard deviation of FA was computed for each tract. In order to fully capture overall tissue alterations induced by pathology at network level, we decided not to separate tissue in lesional and non lesional masks.

#### Evaluation of Network Measures

**FIGURE 2:**
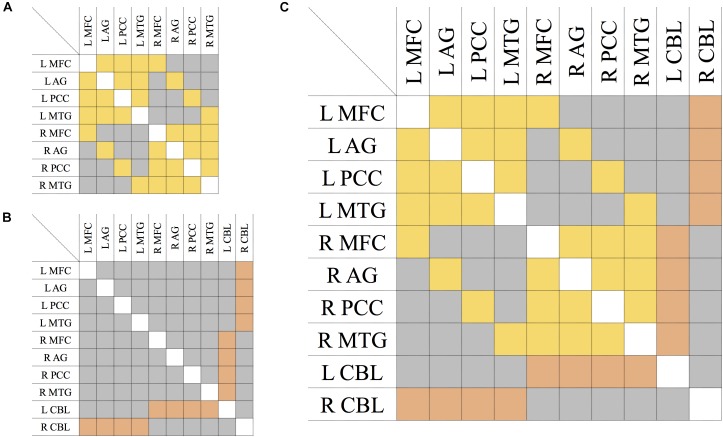
Connectivity matrix of the DMN **(A)**, CBL **(B)**, and CBL-DMN network **(C)**. Symmetric entries of the matrix are shown in yellow and they represent bi-directional bundles of fibers linking network nodes like those tracts connecting DMN regions in the brain cortex. Asymmetric entries are shown in orange and they represent bundles of fibers where the signal is mono-directional like those tracts projecting from the cerebellum to the cerebral cortex. In gray are shown anatomically non-existent connections between nodes and existent connections which are neglected in a specific model, like connections between cortical regions in the model of the cortico-cerebellar loops **(B)**; all of these are numerically represented by zero-elements. The elements along the principal diagonal that would represent self-connections are displayed in white. L/R = left/right. MFC, medial frontal cortex; AG, angular gyrus; PCC, precuneous/posterior cingulate cortex; MTG, middle temporal gyrus; CBL, cerebellum.

Mean FA values were used as link weights in the construction of connectivity matrices, where an entry is the average FA value of the tract connecting the two corresponding nodes (Figure 2). For each participant, the network models were built as follows:

• Fibers linking pairs of cortical regions follow the same pathways, regardless of the directionality of signal transmission. Therefore, the DMN was represented with a symmetric (or undirected) 8 × 8 matrix as shown in Figure 2A.
• Cerebellar input and output signals, instead, follow distinct pathways from and to the brain cortex respectively (cortico-ponto-cerebellar and cerebello-thalamo-cortical pathways). Therefore, cortico-cerebellar loops were modeled with an asymmetric (or directed) 10 × 10 matrix as shown in Figure 2B, where the two rows and columns represent cerebello-thalamo-cortical projections and cortico-ponto-cerebellar projections respectively. All other connections not strictly belonging to the cortico-cerebellar loop (e.g., cortico-cortical connections) are not included within this model.
• The DMN-CBL network model represents the union of the two previous ones (DMN and CBL) and it was obtained by merging the two previous matrices. The result is an asymmetric (or directed) 10 × 10 matrix as depicted in Figure 2C, where the asymmetric elements are only those corresponding to cortico-cerebellar loop tracts.

In all matrices of DMN, CBL, and CBL-DMN models (Figure 2A–C), an empty entry of the matrix represents a tract that is not anatomically plausible or that is not included in the model (as for cortico-cortical connections in CBL).

No FA threshold was applied to connectivity matrices. These were then analyzed with the Brain Connectivity Toolbox (BCT) (Rubinov and Sporns, 2010) as implemented in Matlab (The MathWorks, Inc., Natick, MA, United States^6^). Among all the possible measures provided by network science (Barabási, 2016), GE was chosen as an exemplary measure of the emerging properties of a network. For each subject, the FA-weighted GE was computed for each one of the three network models. Furthermore, the FA average value of the whole WM mask was computed for each subject (WM-FA).

### Statistical Analysis

Statistical analysis was carried out using SPSS (IBM, Armonk, NY, United States^7^). We adopted the standard Tukey’s criterion (interquartile range multiplied by 1.5) to identify outliers (Tukey, 1977).

The following analysis is organized in three main categories: differences (between groups of subjects), associations (between measures within group), and regression (between measures within group).

#### Differences

A general linear model (GLM) was used to ascertain SDMT differences between the two groups of patients (CIMS and CPMS), correcting for EDSS.

*T*-test and one-way ANOVA were performed to assess differences of the following quantities between groups of subjects (*T*-test between HC and MS; ANOVA between HC, CIMS, and CPMS):

• Demographic and clinical data (age, disease duration, EDSS, SDMT, HADS-A, HADS-D, NART);
• MRI metrics (BPF, WM-LL, WM-FA, FA mean values of individual tracts);
• Network measures [GE(DMN), GE(CBL), GE(CBL-DMN)];

*Post hoc* tests (LSD) were run between pairs of groups. The ANOVA was not run to test differences of SDMT scores between CIMS and CPMS that were defined on the basis of SDMT itself or for disease-related measures that were not performed in HC; *T*-tests were, instead, run for the relevant group pairs.

#### Associations

Associations between SDMT and all other measures (demographic, clinical, MRI, and network) were examined computing the Pearson correlation coefficients for HC, MS, CIMS, and CPMS.

Comparisons between correlation coefficients were performed by applying Fisher z-transformation.

In order to assess a possible influence of physical disability on SDMT performance, a partial correlation analysis between SDMT and GE was also performed correcting for EDSS in the MS group and, subsequently, in CIMS and CPMS.

In order to assess the possibility that a bias in FA variability between cortico-cortical and cortico-cerebellar tracts might affect results obtained from our correlation analysis, the mean of the standard deviation of all tracts (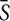), of cortico-cortical tracts (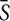
_DMN_ ), and of cortico-cerebellar tracts (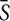
_CBL_ ) were computed for each subject. Partial correlation analysis was then performed between SDMT scores and GE(CBL-DMN) in MS, CIMS, and CPMS groups correcting for 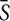, 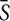
_DMN_, and 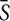
_CBL_ individually.

#### Regression

Values of network GE along with demographic and clinical data (age, EDSS, HADS-A, HADS-D, NART, disease duration) were used in a multiple regression analysis of SDMT variability considering the whole MS population and, subsequently, CIMS and CPMS groups separately.

After that, MRI (BPF, WM-LL, WM-FA), demographic and clinical variables (age, EDSS, HADS-A, HADS-D, NART, disease duration) were individually treated as predictors along with network GE in a multiple regression analysis conducted for the CIMS and CPMS groups to evaluate their contribution to SDMT variation in a multivariable model.

Furthermore, in order to assess the possibility that a bias in FA variability between cortico-cortical and cortico-cerebellar tracts might affect results obtained from our regression analysis, also 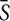
^
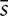
^, 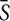
_DMN_, and 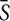
_CBL_ were individually treated as predictors along with network GE in a multivariable model of SDMT.

## Results

The most significant result of this study is that the DMN structural connectivity as assessed by GE explains speed processing impairment in MS subjects, and that in CIMS subjects damage to the extended CBL-DMN is the only predictor of SDMT impairment in a multivariate model, while in CPMS also BPF and EDSS contribute to SDMT scores.

A detailed report of all findings is given here below, starting from a report of tractography results, followed by results of statistical analysis organized in three categories (differences, associations, and regression) as in Section “Statistical Analysis.”

### Tractography

**FIGURE 3:**
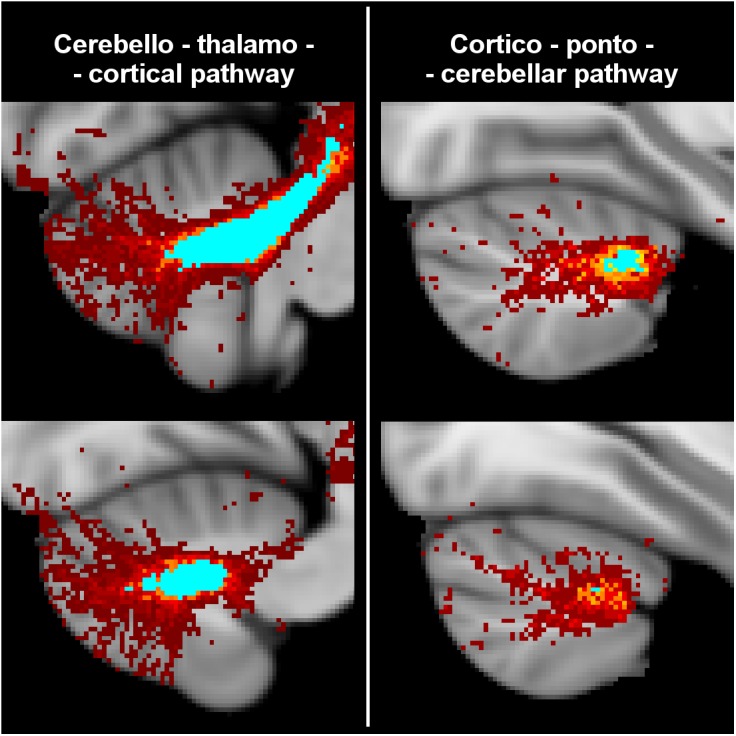
Tractography results from HC were registered to MNI152 standard space at 1 mm resolution to create a population map for each tract. Here are shown the superimposed population maps of the tracts connecting the left hemisphere of the cerebellum to the DMN regions of the right cerebral cortex. On the left are shown cerebello-thlamo-cortical tracts, while on the right are shown cortico-ponto-cerebellar tracts. The voxel color (from dark red to yellow) represents the frequency of occurrence in the specific tract. Light blue regions identify the most consistent part of the tracts, which are common to at least 50% of HC. In the unthresholded population maps, it can be noted that these tracts originate from (cerebello-thalamo-cortical tracts) and project to (cortico-ponto-cerebellar tracts) the posterior lobe of the cerebellum that is associated to cognitive functions.

**FIGURE 4:**
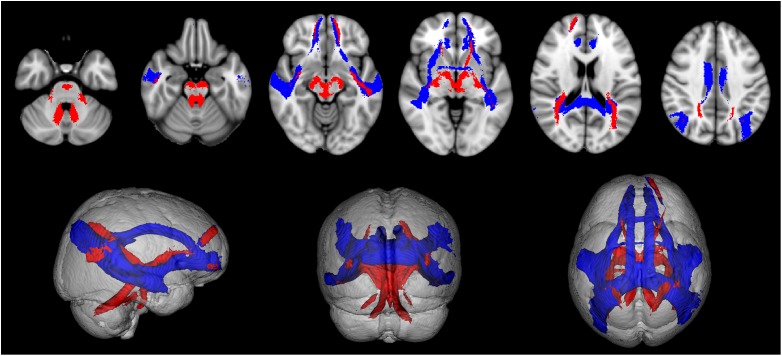
The population map of each tract is thresholded at 50% to select its most consistent part across subjects and to eliminate spurious streamlines. Here are shown all 32 resulting tract masks in MNI 1 mm standard space. These tract masks were subsequently registered to MS patients to compute tract-averaged diffusion FA. Tracts connecting cerebral nodes are displayed in blue, while cortico-cerebellar connections are displayed in red.

All tracts were successfully reconstructed and visually checked by a certified neurologist (MP) for assessment of anatomical plausibility. Figure 3 reports an example of tract population maps (red-yellow color scale) with the resulting thresholded tract masks (light blue). In particular, it shows the superimposition of all tracts involved in cortico-cerebellar loops of the left hemisphere of the cerebellum. It can be noted that these tracts originate from (left: cerebello-thalamo-cortical tracts) and project to (right: cortico-ponto-cerebellar tracts) the posterior lobe of the cerebellum, which is involved in cognitive functions (Habas et al., 2009; Buckner et al., 2011). Figure 4 shows the thresholded masks for all 32 tracts that constitute the CBL-DMN.

### Differences

• One HC outlier was identified on the basis of the distribution of SDMT scores within our HC population. The outlier was removed from subsequent analysis. However, we also ran the analysis including this subject, but this did not significantly change our results and conclusions.
• A summary of the demographic, clinical, MRI, and network variables for each group is given in Table 1 (for one HC and two MS subjects not all tests could be administered).

**Table 1:** Summary of demographic and clinical data and MRI and network variables.

	HC	MS	CIMS	CPMS
Participants (M/F)	10/12	24/44	7/13	16 / 30
Age (years)	36.5 ± 10.8	46.7 ± 11.0	50.3 ± 10.6	45.3 ± 11.0
Disease duration (years)	n.a.	16.7 ± 10.4	21.9 ± 12.6	14.6 ± 8.5
Median EDSS (range)	n.a.	4.5 (1–8.5)	6.0 (1–8.5)	2.0 (1–7.5)
SDMT	62.6 ± 10.8	47.1 ± 12.5	32.1 ± 5.9	53.6 ± 8.3
HADS-A	5.4 ± 4.2	6.6 ± 4.0	6.9 ± 4.0	6.3 ± 3.8
HADS-D	3.0 ± 4.0	5.9 ± 3.6	6.8 ± 3.3	5.4 ± 3.6
NART-predict	108.9 ± 9.3	107.4 ± 10.8	103.2 ± 9.8	109.2 ± 10.8
BPF	0.82 ± 0.02	0.80 ± 0.02	0.79 ± 0.02	0.80 ± 0.02
WM-LL (ml)	n.a.	8.08 ± 9.74	11.85 ± 14.02	6.34 ± 6.72
WM-FA	0.36 ± 0.04	0.24 ± 0.02	0.23 ± 0.03	0.24 ± 0.02
GE(CBL-DMN)	0.34 ± 0.04	0.29 ± 0.03	0.28 ± 0.04	0.29 ± 0.02
GE(DMN)	0.36 ± 0.04	0.29 ± 0.04	0.27 ± 0.04	0.30 ± 0.03
GE(CBL)	0.13 ± 0.02	0.12 ± 0.01	0.11 ± 0.01	0.12 ± 0.01


*Results are shown as mean ± standard deviation unless otherwise reported.*

**FIGURE 5:**
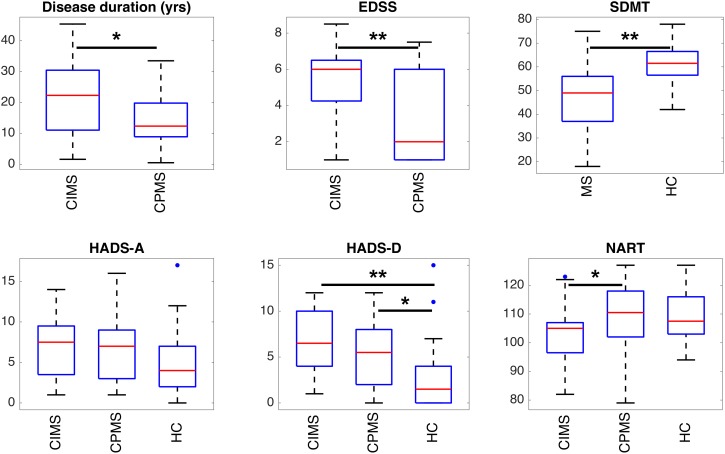
Boxplots representing the summary of clinical scores in the different groups of subjects. Statistically significant differences between groups are indicated with ^∗^*p* < 0.05 and ^∗∗^*p* < 0.01 according to results obtained with ANOVA *post hoc* tests or *T*-tests according to the specific case (see section “Statistical Analysis” and Table 2). It can be observed that SDMT and depression (HADS-D) are significantly different in MS patients and HC. EDSS and, to a lesser extent, disease duration and NART can discriminate between CIMS and CPMS.

**FIGURE 6:**
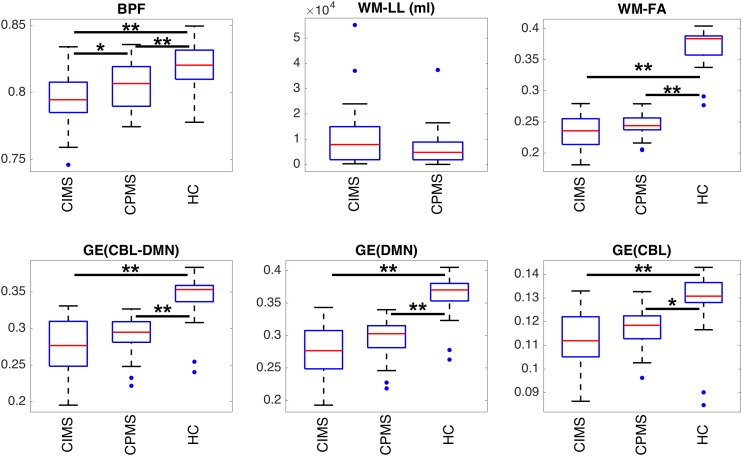
Boxplots representing the summary of MRI metrics in each group of subjects. Statistically significant differences between groups are indicated with ^∗^*p* < 0.05 and ^∗∗^*p* < 0.01 according to results obtained with ANOVA *post hoc* tests or *T*-tests according to the specific case (see section “Statistical Analysis” and Table 2). It can be observed that all measures can distinguish between MS patients and HC. BPF is significantly reduced in CIMS with respect to CPMS. Despite the absence of statistical significance, a similar trend can be observed also for GE and WM-FA.

Significant differences between groups are highlighted in Figure 5, 6 and Table 2. HC controls and MS patients mainly differed in the SDMT and HADS-D scores, while EDSS proved to be the most significantly different measure between CIMS and CPMS. However, the GLM showed that the subdivision of the MS population in CIMS and CPMS based on SDMT scores is significant even when correcting for EDSS (*p* < 0.001).

**Table 2:** *T*-test and one-way ANOVA were used to test differences between groups.

	*T*-test (HC vs. MS)	ANOVA *F* (HC vs. CIMS vs. CPMS)	ANOVA *post hoc*	*T*-test (CIMS vs. CPMS)	*T*-test (HC vs. CIMS, HC vs. CPMS)
Age	^∗∗^	*F*(2,83) = 7.90 ^∗∗^	^∗∗^a,b	/	/
Disease duration	/	/	/	^∗^	/
Median EDSS	/	/	/	^∗∗^	/
SDMT	^∗∗^	/	/	/	^∗∗^ (HC-CI) ^∗∗^ (HC-CP)
HADS-A	n.s.	*F*(2,83) = 0.76 n.s.	n.s.	/	/
HADS-D	^∗∗^	*F*(2,83) = 6.04 ^∗∗^	^∗^b ^∗∗^a	/	/
NART-predict	n.s.	*F*(2,83) = 2.46 n.s.	^∗^c	/	/
BPF	^∗∗^	*F*(2,82) = 9.93 ^∗∗^	^∗^c ^∗∗^a,b	/	/
WM-LL	/	/	/	n.s.	/
WM-FA	^∗∗^	*F*(2,83) = 216.03 ^∗∗^	^∗∗^a,b	/	/
GE(CBL-DMN)	^∗∗^	*F*(2,83) = 27.53 ^∗∗^	^∗∗^a,b	/	/
GE(DMN)	^∗∗^	*F*(2,83) = 37.79 ^∗∗^	^∗∗^a,b	/	/
GE(CBL)	^∗∗^	*F*(2,83) = 10.53 ^∗∗^	^∗^b ^∗∗^a	/	/


*A T-test was first run between HC and MS and results are reported in the first column (^∗∗^*p* < 0.01, ^∗^*p* < 0.05, n.s.: not significant). One-way ANOVA was used to test differences between HC, CIMS, and CPMS. *F-* and *p*-values are reported in the second column, while the third column reports results obtained from ANOVA *post hoc* tests (LSD) between pairs of groups (within this column only significant findings are reported). The ANOVA was not run to test differences of SDMT scores between CIMS and CPMS that were defined on the basis of SDMT itself or for disease-related measures that were not performed in HC; *T*-tests were, instead, run for the relevant group pairs. The fourth column reports results for CIMS-CPMS comparisons of disease-related measures. The last column shows significant differences in SDMT scores between HC and CIMS and between HC and CPMS resulting from *T*-tests. ^*a*^Significant difference between HC and CIMS; ^*b*^Significant difference between HC and CPMS; ^*c*^Significant difference between CIMS and CPMS.*

All MRI measures, and GE in particular, resulted to be significantly different between HC and MS, whereas only BPF showed a significant difference between CIMS and CPMS.

Supplementary Table 1 reports the FA mean value and population standard deviation for each tract in each condition. Statistically significant differences of mean FA values between HC and MS were found for most tracts, while only few of them significantly distinguish between CIMS and CPMS: in particular, the most significant differences were found for those tracts linking the right MTG and AG with the right PCC and for the part of the corpus callosum connecting the left and right AG. FA mean values of each tract are reported for each group also in Supplementary Figure 1, where error bars were obtained by applying the standard propagation of error.

### Associations

**FIGURE 7:**
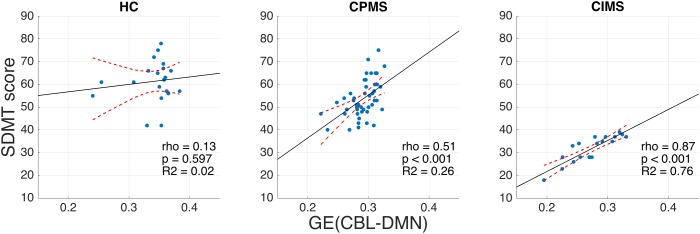
Scatter plots representing SDMT vs. GE (CBL-DMN) for HC **(left)**, CPMS **(center)**, and CIMS **(right)**. Solid black lines represent the linear regression model fit, while dashed red lines represent 95% confidence intervals. It is noteworthy that GE (CBL-DMN) predicts SDMT performance progressively better going from HC (no significant correlation) to CPMS (ρ = 0.51, *p* < 0.001) to CIMS (ρ = 0.87, *p* < 0.001). The difference between correlation coefficients for CPMS and CIMS is statistically significant (*p* < 0.01), as verified by applying Fisher z-transformation.

No significant correlation between SDMT scores and MRI, demographic or clinical variables was observed in HC (Table 3). In MS, however, highly significant correlations (ρ ≈ 0.5 and *p* < 0.001) were found for FA-weighted metrics, EDSS and BPF and significant correlations were also found for age, disease duration and WM-LL, although with lower coefficients (ρ ≈ 0.3 and *p* < 0.01). In the CIMS group: the strongest correlations with SDMT performance were found for GE(CBL-DMN), GE(DMN), and GE(CBL) (ρ > 0.8 and *p* < 0.001); significant and high correlations were also found for WM-FA and BPF; no significant correlation was found between SDMT and EDSS. In the CPMS group: BPF showed the best association with SDMT performance (ρ = 0.57 and *p* < 0.001); significant correlations were found also for GE(CBL-DMN), GE(DMN), GE(CBL), and EDSS. Figure 7 graphically shows the association between SDMT and GE(CBL-DMN) for HC, CIMS, and CPMS.

**Table 3:** Correlations of MRI measures and clinical data with SDMT.

	HC		MS		CIMS		CPMS	
GE(CBL-DMN)	0.13 (0.597)	0.02	**0.54 (<0.001)**	**0.29**	**0.87 (<0.001)**	**0.76**	**0.51 (<0.001)**	**0.26**
GE(DMN)	0.15 (0.524)	0.02	**0.57 (<0.001)**	**0.33**	**0.82 (<0.001)**	**0.67**	**0.48 (0.001)**	**0.23**
GE(CBL)	0.07 (0.785)	<0.01	**0.53 (<0.001)**	**0.28**	**0.80 (<0.001)**	**0.64**	**0.52 (<0.001)**	**0.27**
WM-FA	0.16 (0.508)	0.02	**0.47 (<0.001)**	**0.22**	**0.73 (<0.001)**	**0.53**	0.37 (0.012)	0.13
Age	–0.09 (0.692)	0.01	–**0.32 (0.009)**	**0.10**	–0.17 (0.469)	0.03	–0.28 (0.056)	0.08
EDSS	n.a.	n.a.	–**0.52 (<0.001)**	**0.27**	–0.04 (0.861)	<0.01	–**0.46 (0.001)**	**0.21**
Disease duration	n.a.	n.a.	–**0.35 (0.004)**	**0.12**	–0.27 (0.246)	0.07	–0.12 (0.416)	0.02
WM-LL	n.a.	n.a.	–**0.39 (0.002)**	**0.15**	–0.45 (0.055)	0.20	–0.31 (0.050)	0.09
BPF	–0.13 (0.599)	0.02	**0.55 (<0.001)**	**0.31**	**0.63 (0.004)**	**0.39**	**0.57 (<0.001)**	**0.33**
NART-predict	0.04 (0.873)	<0.01	0.32 (0.010)	0.10	0.21 (0.376)	0.04	0.18 (0.221)	0.03
HADS-A	0.23 (0.330)	0.05	–0.09 (0.476)	0.01	–0.17 (0.469)	0.03	–0.03 (0.872)	<0.01
HADS-D	0.09 (0.695)	0.01	–0.30 (0.016)	0.09	–0.29 (0.220)	0.08	–0.24 (0.109)	0.05


*Person correlation coefficients are reported along with *p*-values (in brackets) and the corresponding *R*^2^ (each second column). Correlations with *p*-value < 0.01 are highlighted in bold.*

In the CIMS group, GE(DMN) and GE(CBL) explained 67 and 64% of SDMT variance respectively, while GE(CBL-DMN) increased this value to 76%. However, in the CPMS group network GE could explain only 23% (DMN), 27% (CBL), and 26% (CBL-DMN) of the SDMT variability (Table 3).

Since the CBL-DMN represents the most comprehensive model and since the results it provided in both CIMS and CPMS were better than or similar to those obtained with DMN and CBL separately (Table 3), we considered only GE(CBL-DMN) for the following partial correlation analysis and, later, for regression analysis.

Partial correlation analysis showed that the statistical significance of the correlation between SDMT and GE(CBL-DMN) is preserved within each group of patients also when controlling for EDSS, 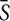, 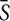
_DMN,_ or 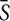
_CBL_ (Supplementary Table 2).

### Regression

The multivariable regression analysis of SDMT variability that considered GE(CBL-DMN) and clinical scores (age, EDSS, HADS-A, HADS-D, NART, disease duration) as explanatory variables in a single model showed that this model was statistically significant within the whole MS group (*p* < 0.001, *R*^2^ = 0.51, *R*^2^ corrected = 0.45) and also within CIMS (*p* = 0.002, *R*^2^ = 0.81, *R*^2^ corrected = 0.70) and CPMS (*p* = 0.002, *R*^2^ = 0.43, *R*^2^ corrected = 0.32) subgroups. In particular, significant contributions were provided by GE(CBL-DMN) (*p* = 0.002), NART scores (*p* = 0.007), and EDSS scores (*p* = 0.034) for MS, while GE(CBL-DMN) resulted the only significant predictor of SDMT variability for both CIMS and CPMS (*p* < 0.001 and *p* = 0.004 respectively).

Multivariable models of SDMT variability built with pairs of explanatory variables, where GE(CBL-DMN) is tested against each clinical and MRI variable, showed that in CIMS GE(CBL-DMN) remained the only significant predictor of the SDMT variability (Table 4). However, for CPMS patients EDSS and BPF also contributed to the model by increasing the *R*^2^ value from 0.26 obtained with the only GE(CBL-DMN) to 0.34 and 0.37 respectively.

**Table 4:** Linear regression analysis in CIMS and CPMS patients.

	Model predictors	*R*	*R*^2^	*R*^2^ corrected	Significance of GE(CBL-DMN)	Significance of the other variable
CIMS	GE(CBL-DMN), Age	0.87	0.76	0.74	<0.001	0.793
	GE(CBL-DMN), EDSS	0.87	0.76	0.74	<0.001	0.851
	GE(CBL-DMN), Disease duration	0.87	0.76	0.74	<0.001	0.931
	GE(CBL-DMN), WM-LL	0.88	0.77	0.74	<0.001	0.630
	GE(CBL-DMN), WM-FA	0.88	0.78	0.75	<0.001	0.326
	GE(CBL-DMN), BPF	0.88	0.78	0.75	<0.001	0.634
	GE(CBL-DMN), NART-predict	0.87	0.76	0.74	<0.001	0.804
	GE(CBL-DMN), HADS-A	0.87	0.76	0.74	<0.001	0.847
	GE(CBL-DMN), HADS-D	0.88	0.78	0.75	<0.001	0.296
CPMS	GE(CBL-DMN), Age	0.57	0.32	0.29	<0.001	0.063
	GE(CBL-DMN), EDSS	0.59	0.34	0.31	0.004	0.025
	GE(CBL-DMN), Disease duration	0.51	0.26	0.23	<0.001	0.867
	GE(CBL-DMN), WM-LL	0.52	0.27	0.23	0.004	0.944
	GE(CBL-DMN), WM-FA	0.51	0.26	0.23	0.009	0.990
	GE(CBL-DMN), BPF	0.61	0.37	0.35	0.087	0.008
	GE(CBL-DMN), NART-predict	0.53	0.28	0.25	<0.001	0.319
	GE(CBL-DMN), HADS-A	0.51	0.26	0.23	<0.001	0.813
	GE(CBL-DMN), HADS-D	0.54	0.29	0.26	<0.001	0.192


*Symbol digit modalities test is considered as the dependent variable and GE(CBL-DMN) is tested against each other MRI or clinical variable. The second last column reports the *p*-values associated with GE(CBL-DMN) within each model and the last column reports the *p*-value associated with each second predictor. GE(CBL-DMN) holds as the only significant predictor against each other variable in CIMS, while in CPMS EDSS and BPF are also significant predictors of SDMT performance.*

Multivariable regression analysis of SDMT variability performed considering GE(CBL-DMN) and individual measures of FA variability of tracts (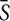, 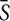
_DMN__,_ and 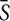
_CBL_) as explanatory variables showed that none of these last variables provides a significant contribution to the model in any group of patients.

## Discussion

In this study we demonstrate that diffusion FA and GE of the DMN-CBL network better explains cognitive performance of MS compared to GE of DMN or CBL on their own; this confirms the importance of the overall structural integrity of the extended DMN-CBL in supporting healthier cognition as measured by SDMT. Indeed, in CIMS patients the DMN structural GE emerges already as the best predictor of cognitive performance with respect to other MRI metrics and clinical scores, and extending the DMN to include the cerebellar nodes increases further the strength of such association. Interestingly, in CPMS patients SDMT performance is better explained by brain atrophy and, to a lesser extent, disability. The greater association between GE and SDMT in CIMS compared with CPMS is consistent with the concept of network collapse, as proposed by Schoonheim et al. (2015), which would be very interesting to confirm with longitudinal studies.

Multiple sclerosis has multiple effects on brain tissue including hindered neuronal communication, which has been already proposed as a key mechanism for the development of cognitive impairment in MS (Dineen et al., 2009; Bozzali et al., 2013): results from regional observations in MS indicate that when the structural integrity of DMN WM bundles (as assessed, for example, by diffusion tensor metrics) is compromised, this reflects on cognitive performance (Roosendaal et al., 2009, 2010; Rocca et al., 2010; Hawellek et al., 2011; Zhou et al., 2014). However, damage in MS is highly subject-specific and evidence of alterations of the tissue diffusion properties of individual WM bundles could provide only modest correlations.

Therefore, it became clear that a local approach is not sufficient to describe the decline of a specific cognitive domain. Moreover, given the nature of the MS damage and the interactive behavior of brain networks, one could hypothesize that different subjects with different portions of the DMN structure affected, may even result having a similar global derangement.

The level of alteration of a network can be assessed through the application of network science methods: when applied to brain networks they can summarize local damage affecting different tracts linking network nodes in a number of comprehensive metrics. Network science has already been applied to study brain networks topology in MS at different scales (He et al., 2009; Shu et al., 2011, 2016; Pardini et al., 2015; Llufriu et al., 2017), thus proving that network metrics can capture MS-induced abnormalities and outperform non-network-based MRI measures in the prediction of disability (Pardini et al., 2014, 2015).

Brain functions are thought to be supported by specific sub-networks and, therefore, the structural pathological correlates of specific impaired functions are expected to lie in the corresponding sub-network (Menon, 2011; Griffa et al., 2013). Hence, this work focused on the DMN, a sub-network that has been associated with information processing speed (Rocca et al., 2010; Sumowski et al., 2010; Forn et al., 2013; Bonavita et al., 2015), rather than addressing the whole-brain network, which may be more appropriate to characterize the macroscopic patterns of the disease. Moreover, the whole-brain network approach may not be sufficiently specific to link the clinical performance and the structural alterations induced by MS (Cercignani and Gandini Wheeler-Kingshott, 2018).

Within this framework, several metrics could be used. Indeed, GE was chosen as it is a measure of network integration, thus capturing overall network damage and evaluating the efficiency of communication between distant regions of the DMN connected by long association fibers. Directed nodal strength would convey very similar results as GE (Supplementary Table 5), while betweenness centrality (which is another local network measure that assumes discrete values) would result in very skewed data when assessed over a limited number of nodes like in our case, hence loosing sensitivity to subtle differences in pathological presentations (Supplementary Table 4). Similarly, several edge-weights could have been chosen for this work. Mean FA was considered most favorably as link weight because of its recognized sensitivity to MS-induced damage in both focal lesions and NAWM (Werring et al., 1999; Kutzelnigg et al., 2005). The widespread microstructural damage affecting NAWM has been linked to disability in MS (Dineen et al., 2009; Hulst et al., 2013; Deppe et al., 2015); moreover, such damage is independent of WM lesion location and, in the framework of connectomics, it could impact the function of networks as much as focal lesions (Cercignani and Gandini Wheeler-Kingshott, 2018). Our results indeed suggest that FA-weighted GE captures MS-related damage affecting the DMN circuit and that it significantly correlates with SDMT performance in MS. Consistently with previous studies, we found that other measures like WM-FA, BPF and EDSS also contribute to SDMT scores (Louapre et al., 2014; Deppe et al., 2015), suggesting that altered microstructure, atrophy and the disease course influence cognition in MS. It remains to be discussed whether the DMN connectivity to the cerebellum can further strengthen the results.

The cerebellum has been shown to have an impact on MS-related cognitive impairment (Weier et al., 2014, 2015): in particular, a link has been established between information processing speed deficit, posterior lobules of the cerebellum and cerebellar peduncles (Forn et al., 2011; Moroso et al., 2017a,b). Moreover, it has been hypothesized that the alteration of the cortico-cerebellar pathways supporting automation and attention processes may lead to poor cognitive performance (Valentino et al., 2009; Bonnet et al., 2010). Our correlation analysis shows that GE(CBL-DMN) contribution to SDMT variance is greater than (in CIMS) or comparable to (in CPMS) the contribution provided by GE(DMN) or GE(CBL) alone. In particular, the inclusion of the cerebellum and of cortico-cerebellar tracts increased the explained SDMT variance R^2^ from 67 and 64% respectively obtained with GE(DMN) and GE(CBL) to 76% in CIMS patients, while GE(CBL-DMN), GE(DMN), and GE(CBL) provided almost equal contribution to SDMT variance for CPMS, respectively *R*^2^ = 0.26, *R*^2^ = 0.23, and *R*^2^ = 0.27. This indicates that the DMN network connectivity with the cerebellum, rather than the cortical or the cerebellar pathology *per se*, is functionally relevant to SDMT performance in MS and highlights the need to address networks as a whole rather than focusing on separate components (see also Supplementary Table 3). Furthermore, these results also point to the fact that the role of specific networks may evolve during the course of a disease: the DMN, in fact, seems to have a different weight on cognitive performance at different stages of the disease. In patients with preserved cognitive functions (e.g., in CPMS), the damage accumulated since the disease onset might affect the DMN network partially, hence preserving its functioning. When presenting with worse cognitive functions (e.g., as in CIMS), captured by a worse SDMT score, the accumulated structural damage to the DMN reaches a critical threshold, beyond which the network functioning collapses and the cognitive performance worsens more rapidly (Schoonheim et al., 2015; Shu et al., 2016; Cercignani and Gandini Wheeler-Kingshott, 2018).

Here, interestingly it is to notice that *per se* GE(CBL-DMN) is not significantly different when comparing directly CIMS and CPMS, result which might depend on a number of factors, including differences in group size (Figure 6). FA variability along tracts may represent a possible confounding factor. However, partial correlation and regression analysis showed that tract FA variability, as expressed by 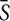, 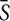
_DMN__,_ and 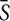
_CBL_, does not drive the different trend of associations between SDMT and GE observed in CIMS and CPMS. The CIMS and CPMS groups have also significantly different median EDSS, although the EDSS range is comparable between both groups and no correlation was found between SDMT and EDSS scores in CIMS. WM-FA and GE(CBL-DMN) are key factors of the poor SDMT performance in CIMS patients, with unmatched ρ correlation coefficients ranging respectively from 0.73 to 0.87 (*p* < 0.001). The network efficiency alone provided *R*^2^ = 0.76 and linear regression analysis showed that GE(CBL-DMN) is the best predictor of poor cognitive performance, while no other variable adds a relevant contribution to the percentage of the explained SDMT variance. However, in CPMS patients, BPF and EDSS have also got a positive effect on SDMT performance, as shown by results of the linear regression model that includes these variables as well as GE; such results may be interpreted by stating that SDMT performance in CPMS patients is driven by both a “milder” alteration of the network efficiency and the general disease course captured by EDSS and BPF. Therefore, we believe this defines a possible distinct role of the CBL-DMN network in SDMT performance at different cognitive stages. In particular, our results show that the association between GE(CBL-DMN) and SDMT performance is progressively strengthened with increasing network derangement. In keeping with this statement, in HC the CBL-DMN structural integrity as assessed by GE(CBL-DMN) is untouched and the SDMT performance is completely unrelated to GE; in CPMS, the SDMT scores are only partially dependent on GE(CBL-DMN) as other factors associated with the general disease course contribute to performance; in CIMS the association between GE(CBL-DMN) and SDMT scores becomes much stronger (Figure 7) implying that CBL-DMN assumes a more prominent role in cognitive performance.

### Limitations

Our work is not without limitations. Here, we report an important association between GE(CBL-DMN) and SDMT performance in MS, but this is a cross-sectional study, so causality between network microstructural damage and SDMT worsening can only be hypothesized and a longitudinal study is warranted in the future.

Network science provides many different measures and in brain network studies several different link weights can be used, but it is not clear yet which combination could be the best to capture clinically relevant network abnormalities in MS. Here, we chose FA-weighted GE as an exemplary measure to capture the overall network damage. We also addressed local network measures like the directed nodal strength and the betweenness centrality, but these measures did not provide further information to our results (Supplementary Tables 4, 5). Further work is required to clarify which network measures are most sensitive to pathological damage and which are most associated to MS-related impairment.

With regards to link weights, the diffusion tensor model that we used has been shown to be sensitive but not-specific to the different pathological substrates of MS (Cercignani and Gandini Wheeler-Kingshott, 2018). Alternative frameworks, like model-free approaches or higher-order diffusion models, could provide metrics more specific to pathology-related microstructural damage: these metrics combined with network measures could give a better insight into the mechanisms leading from pathology alterations at the cellular level to subsequent functional impairment.

In the present study we only used one measure (FA) to weight DMN connection, but multimodal approaches using different imaging modalities, for example combining different structural measures (Pardini et al., 2015) or structural and functional imaging (Zhou et al., 2014; Romascano et al., 2015) have been proposed and may further improve our ability to explain cognitive outcomes. This could be scoped in future work and composite weights could be adopted.

Finally, a single neuropsychological test (the SDMT) was here used to divide the MS population in CIMS and CPMS groups of patients. The SDMT was tested against extensive batteries of neuropsychological tests developed to detect cognitive impairment in MS and it provided very promising results (Parmenter et al., 2007; Van Schependom et al., 2014). However, it is also clear that a single test cannot capture disability in all cognitive domains and further studies are demanded to validate results presented here considering more extensive assessments of patients’ cognitive status.

## Conclusion

We found that DMN structural GE [in particular GE (CBL-DMN)] is reduced in MS and this is increasingly linked with SDMT performance as cognitive impairment becomes apparent. Concurrently, with increasing cognitive impairment, GE(CBL-DMN) dominates over more global measures of brain pathology (WM-FA and BPF) or disability (EDSS). We showed that connections between the cerebellum and the DMN are relevant to SDMT performance: cortico-cerebellar connections within the DMN play an important role in the network organization and their MS-related structural alteration affects cognitively-relevant network functioning. These results warrant future longitudinal studies to assess the clinical translation potential of network measures for the early detection of cognitive processing speed decline.

## Author Contributions

GS, MP, and CGW-K conceptualized and designed the study. GS performed the data analysis and the statistical analysis with the contribution of MP and GC. MP and CGW-K supervised the study. DC, ED’A, and AL provided support and guidance with data interpretation. GS, CGW-K, and DC drafted the manuscript. All authors critically revised the work and contributed to the final version of the manuscript.

## Conflict of Interest Statement

DC has received honoraria (paid to his employer) from Ismar Healthcare NV, Swiss MS Society, Excemed (previously Serono Symposia International Foundation), Merck, Bayer, and Teva for faculty-led education work; Teva for advisory board work; meeting expenses from Merck, MS Trust, National MS Society, Novartis, Société des Neurosciences and Teva; and has previously held stock in GlaxoSmithKline. MP receives research support from Novartis and honoraria for the participation in advisory board meetings activities from Merck. The remaining authors declare that the research was conducted in the absence of any commercial or financial relationships that could be construed as a potential conflict of interest.

## Footnotes

1 http://www.fmrib.ox.ac.uk/fsl/

2 http://www.mrtrix.org

3 http://cmictig.cs.ucl.ac.uk/wiki/index.php/NiftyReg

4 http://www.xinapse.com

5 http://www.fil.ion.ucl.ac.uk/spm

6 https://www.mathworks.com

7 https://www.ibm.com

## Abbreviations

BPF: brain parenchymal fraction
CBL-DMN: extended DMN including cerebellar nodes
CIMS: cognitively impaired MS patients
CPMS: cognitively preserved MS patients
EDSS: expanded disability status scale
FA: fractional anisotropy
GE: global efficiency of a network
GE(…): global efficiency of the specified network
HADS: hospital anxiety and depression scale
NART: national adult reading test
NAWM: normal-appearing white matter
SDMT: symbol digit modalities test
TDI: track density imaging
WM-FA: white matter average FA
WM-LL: white matter lesion load
