# Supplementary material for: Default Mode Network Structural Integrity and Cerebellar Connectivity Predict Information Processing Speed Deficit in Multiple Sclerosis
Source: Front Cell Neurosci. 2019 Feb 11;13:21. doi: 10.3389/fncel.2019.00021 (PMC6396736; doi:10.3389/fncel.2019.00021)
Supplement: Supplementary file 1 [file Data_Sheet_1.PDF]

Supplementary Material

Default mode network structural integrity and cerebellar connectivity predict information processing speed deficit in multiple sclerosis

Giovanni Savini[1*], Matteo Pardini[2,3], Gloria Castellazzi[4,5], Alessandro Lascialfari[1], Declan Chard[5,6], Egidio D'Angelo[7,8], Claudia AM Gandini Wheeler-Kingshott[5,7,9]

*** Correspondence:**
Giovanni Savini
giovanni.savini@unimi.it

1 Supplementary Figures and Tables

Supplementary Table 1:

Mean FA value of individual tracts of the network in each group. Results are shown as mean ± population standard deviation. Statistical significance expressed by p-values refers to the values reported in the two columns at the left (* p<0.05, ** p<0.01)

	HC	MS	p	CIMS	CPMS	p
**Cortical tracts**						
L MTG - L MFC	0.40 ± 0.05	0.34 ± 0.04	**	0.33 ± 0.05	0.34 ± 0.03	
L MTG - L AG	0.44 ± 0.02	0.34 ± 0.04	**	0.34 ± 0.07	0.34 ± 0.03	
L MTG - L PCC	0.44 ± 0.04	0.35 ± 0.05	**	0.33 ± 0.07	0.35 ± 0.04	*
L MFC - L AG	0.43 ± 0.04	0.38 ± 0.04	**	0.37 ± 0.06	0.38 ± 0.03	
L MFC - L PCC	0.39 ± 0.04	0.31 ± 0.06	**	0.33 ± 0.06	0.30 ± 0.05	
L AG - L PCC	0.42 ± 0.03	0.31 ± 0.04	**	0.30 ± 0.06	0.32 ± 0.03	
R MTG - R MFC	0.41 ± 0.06	0.35 ± 0.03	**	0.34 ± 0.04	0.35 ± 0.03	
R MTG - R AG	0.43 ± 0.03	0.33 ± 0.05	**	0.31 ± 0.06	0.34 ± 0.04	
R MTG - R PCC	0.44 ± 0.03	0.35 ± 0.05	**	0.32 ± 0.07	0.36 ± 0.04	**
R MFC - R AG	0.44 ± 0.04	0.38 ± 0.04	**	0.37 ± 0.05	0.38 ± 0.03	
R MFC - R PCC	0.38 ± 0.04	0.28 ± 0.06	**	0.29 ± 0.06	0.28 ± 0.05	
R AG - R PCC	0.42 ± 0.04	0.32 ± 0.05	**	0.29 ± 0.06	0.33 ± 0.03	**
L MTG - R MTG	0.41 ± 0.08	0.33 ± 0.05	**	0.32 ± 0.05	0.34 ± 0.04	
L MFC - R MFC	0.56 ± 0.12	0.50 ± 0.07	**	0.49 ± 0.09	0.51 ± 0.06	
L AG - R AG	0.52 ± 0.06	0.45 ± 0.07	**	0.41 ± 0.08	0.46 ± 0.05	**
L PCC - R PCC	0.61 ± 0.10	0.46 ± 0.07	**	0.43 ± 0.09	0.48 ± 0.06	*

**Cortico-cerebellar tracts**						
L MTG to R CBL	0.38 ± 0.07	0.34 ± 0.04	**	0.33 ± 0.05	0.35 ± 0.04	
L MFC to R CBL	0.41 ± 0.10	0.39 ± 0.05		0.38 ± 0.06	0.40 ± 0.05	
L AG to R CBL	0.43 ± 0.05	0.38 ± 0.04	**	0.37 ± 0.05	0.38 ± 0.03	
L PCC to R CBL	0.43 ± 0.06	0.36 ± 0.04	**	0.35 ± 0.04	0.37 ± 0.03	
R MTG to L CBL	0.39 ± 0.07	0.36 ± 0.04	*	0.35 ± 0.06	0.36 ± 0.04	
R MFC to L CBL	0.43 ± 0.08	0.40 ± 0.04	*	0.40 ± 0.05	0.40 ± 0.04	
R AG to L CBL	0.45 ± 0.05	0.40 ± 0.04	**	0.38 ± 0.05	0.41 ± 0.03	*
R PCC to L CBL	0.46 ± 0.06	0.40 ± 0.04	**	0.38 ± 0.05	0.41 ± 0.04	*
**Cerebello-cortical tracts**						
R CBL to L MTG	0.42 ± 0.05	0.38 ± 0.03	**	0.37 ± 0.04	0.38 ± 0.03	
R CBL to L MFC	0.38 ± 0.04	0.34 ± 0.03	**	0.34 ± 0.03	0.35 ± 0.02	
R CBL to L AG	0.41 ± 0.02	0.37 ± 0.03	**	0.36 ± 0.05	0.37 ± 0.03	
R CBL to L PCC	0.40 ± 0.05	0.37 ± 0.03	**	0.36 ± 0.03	0.37 ± 0.03	
L CBL to R MTG	0.42 ± 0.04	0.38 ± 0.04	**	0.37 ± 0.04	0.38 ± 0.03	
L CBL to R MFC	0.39 ± 0.05	0.36 ± 0.03	**	0.35 ± 0.03	0.36 ± 0.02	
L CBL to R AG	0.40 ± 0.03	0.38 ± 0.03	**	0.36 ± 0.04	0.38 ± 0.03	*
L CBL to R PCC	0.39 ± 0.04	0.38 ± 0.04		0.36 ± 0.04	0.38 ± 0.03	*

Supplementary Table 2:

Partial correlation analysis between SDMT scores and GE(CBL-DMN) values controlling for confounding variables shown in the left column. Correlation coefficients are reported along with p-values (in brackets) for each group of patients.

SDMT - GE(CBL-DMN) partial correlation		**MS**	**CIMS**	**CPMS**
Correcting for	EDSS	0.46 (<0.001)	0.87 (<0.001)	0.42 (0.004)
	$\bar{s}$	0.53 (<0.001)	0.85 (<0.001)	0.54 (<0.001)
	$\bar{s}_{CBL}$	0.56 (<0.001)	0.87 (<0.001)	0.52 (<0.001)
	$\bar{s}_{DMN}$	0.46 (<0.001)	0.83 (<0.001)	0.56 (<0.001)

Supplementary Table 3:

Correlation analysis between SDMT scores and the GE of the network obtained by removing one of the key regions of the DMN; in this case the left and right MTG nodes and related tracts were removed from the CBL-DMN network model, resulting in an 8x8 connectivity matrix. Correlation coefficients are reported for each group along with p-values (in brackets). It is to notice that the correlation of network GE with SDMT scores remains unaltered. This supports the argument that the increased correlation obtained when including the CBL is not due to a higher number of regions, but rather to the specific role of the cerebellum.

**HC**		**MS**		**CIMS**		**CPMS**	
0.12 (0.609)	0.01	0.52 (<0.001)	0.27	0.88 (<0.001)	0.77	0.48 (0.001)	0.23

Supplementary Table 4:

Betweenness centrality (BC) is a local network measure. However, BC is a discrete measure and it results in very skewed data when assessed over a limited number of nodes like in our case, hence loosing sensitivity to subtle differences in pathological presentations. This table of frequencies shows for each node the number of subjects for whom BC assumes the reported discrete values (first column).

**BC**	**L MFC**	**L AG**	**L PCC**	**L MTG**	**R MFC**	**R AG**	**R PCC**	**R MTG**	**L CBL**	**R CBL**
**0,000**	1				1				87	88
**0,014**	1			5				3	1	
**0,028**				78	1	4		84		1
**0,042**	1			1			1	1	1	1
**0,056**	9	45	14	4	15	30	14	1	1	
**0,069**	1	6	1		3	2	2	1		
**0,083**	14	23	23	2	24	30	25			
**0,097**	3	7	11		10	9	12			
**0,111**	20	6	11		13	8	18			
**0,125**	12		8		5	2	5			
**0,139**	26		18		17	4	10			
**0,153**	1		3				3			
**0,167**	1				1					
**0,181**		2	1			1				
**0,194**		1								

Supplementary Table 5:

Correlation analysis between SDMT scores and values of directed nodal strength (dNS) for each node. Correlation coefficients are reported for each group of subjects along with p-values (in brackets). It is to notice that correlation coefficients within a group of subjects are rather homogeneous across nodes and that these values are close to that obtained with GE. This indicates that GE well summarizes the information provided by dNS, which is a local network measure. This supports our choice to consider GE as our exemplary network measure.

	**HC**	**MS**	**CIMS**	**CPMS**
GE(CBL-DMN)	0.13 (0.597)	0.54 (<0.001)	0.87 (<0.001)	0.51 (<0.001)
L MFC	0.12 (0.612)	0.45 (<0.001)	0.84 (<0.001)	0.57 (<0.001)
L AG	0.04 (0.870)	0.48 (<0.001)	0.83 (<0.001)	0.45 (0.002)
L PCC	0.16 (0.508)	0.47 (<0.001)	0.82 (<0.001)	0.44 (0.002)
L MTG	0.13 (0.579)	0.49 (<0.001)	0.81 (<0.001)	0.57 (<0.001)
R MFC	0.18 (0.451)	0.45 (<0.001)	0.85 (<0.001)	0.53 (<0.001)
R AG	0.10 (0.673)	0.55 (<0.001)	0.84 (<0.001)	0.38 (0.009)
R PCC	0.15 (0.537)	0.52 (<0.001)	0.79 (<0.001)	0.37 (0.011)
R MTG	0.16 (0.494)	0.56 (<0.001)	0.86 (<0.001)	0.50 (<0.001)
L CBL	0.11 (0.645)	0.51 (<0.001)	0.79 (<0.001)	0.45 (0.002)
R CBL	0.02 (0.948)	0.52 (<0.001)	0.79 (<0.001)	0.53 (<0.001)

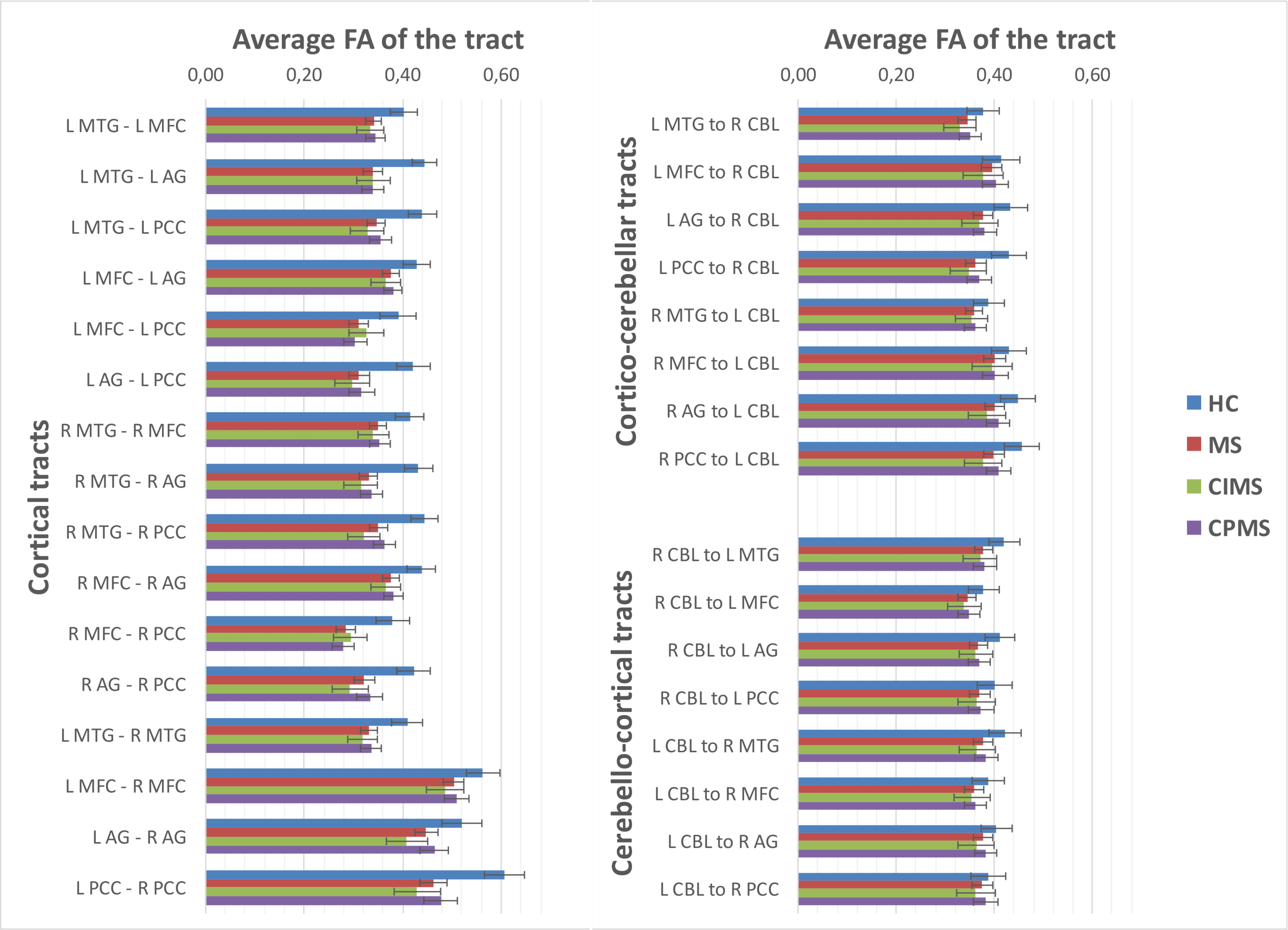

Supplementary Figure 1. Mean FA value of individual tracts of the network displayed for each group of subjects. Error bars represent the standard deviation of the tract obtained by applying the standard propagation of error. On the left are shown results for tracts linking nodes of the brain cortex, while on the right are shown results for tracts linking the cerebellum with regions of the brain cortex.
